# The ESR Patient Advisory Group (ESR-PAG)

**DOI:** 10.1007/s13244-015-0395-z

**Published:** 2015-03-17

**Authors:** European Society of Radiology (ESR)

**Affiliations:** Neutorgasse 9/2, 1010 Vienna, Austria

**Keywords:** Patient/doctor communication, Patient organisations, Patient-centred radiology, Personalised radiology, ESR activities

## Abstract

**Abstract:**

The European Society of Radiology (ESR) Patient Advisory Group addresses the issue of communication among radiologists, radiographers and their patients. Initiated in 2013, the group represents many patient groups, working together with the ESR to raise awareness of medical imaging amongst patients, improve patient knowledge about imaging procedures, liaise on policy issues of common interest, involve patient representatives in strategic decisions regarding medical imaging and ensure that a patient-centred, “human” approach is embedded in the structure of the ESR. The ESR strongly believes that close collaboration with patient organisations is beneficial for both stakeholders, helping radiologists to understand patients’ needs, to adapt their practice accordingly and to improve their communication skills. These factors are important to help radiologists find acknowledgement as clinicians and overcome the specialty’s poor visibility. Close collaboration with professionals will allow patients to obtain a better understanding of the work of everyone involved in a radiological department, of the advantages and limitations of imaging, and what to expect from a radiological examination or an imaging-guided interventional procedure. Thus, this collaborative effort is not simply limited to improving patient satisfaction; the ultimate goal is to enhance the faith and trust that patients place in the medical imaging profession and all the radiology professionals involved.

***Main messages*:**

• *Good communication between patient and doctor is important for proper patient care.*

• *The ESR Patient Advisory Group specifically addresses this communication issue.*

• *The activities aim at developing a reciprocal relationship between radiologists/radiographers and patients.*

• *Both radiologists/radiographers and patients need to be “educated” to improve communication.*

“It has long been recognised that difficulties in the effective delivery of healthcare can arise from problems in communication between patient and provider rather from any failing in the technical aspects of medical care” [1]. This statement published in the American College of Physicians Journal Club in 2002 reminds us that, in medical practice, good communication between patient and doctor is a key factor for proper diagnosis, treatment and patient satisfaction. The same holds true in the case of communication between patients and those healthcare workers (e.g. nurses, radiographers) who are in constant face-to-face contact with patients [2, 3].

The European Society of Radiology (ESR) believes that this also applies for the radiology profession, and that there is a need to improve communication between radiology professionals and patients. With this in mind, the ESR has created a “Patient Advisory Group” (ESR-PAG) to address the issue of communication between radiology professionals (radiologists, radiographers, residents, nurses, administrators, etc.) and their patients.

The concept of initiating the ESR-PAG arose as a result of the good collaboration developed during the “Alliance for MRI” campaign, in which the combined efforts of patient organisations and the scientific community in European Affairs proved crucial for a successful result. The official launch took place during the European Congress of Radiology (ECR) 2013 after a series of preparatory meetings with patient group representatives, held during the autumn of 2012.

After the first face-to-face meeting in Vienna, as well as during a teleconference on 15 April 2013, the specific aims of the group were identified. They can be summarised in six points:

- Improve communication between radiologists and patients
- Raise awareness about medical imaging amongst patients
- Improve knowledge of patients about imaging procedures
- Liaise with patient groups on policy issues of common interest
- Involve patient representatives in strategic decisions regarding medical imaging
- Ensure a patient-centred, “human” approach, embedded in the work of ESR

Since then, the ESR-PAG has been active for almost 2 years and has already convened several times: there were two face-to-face meetings in Vienna during the ECR 2013 and ECR 2014; however, most of the business has been managed through teleconferences. The patient groups involved in this initiative are currently:

- The European Patients’ Forum (EPF—Nicola Bedlington, Secretary General)
- The European Federation of Crohn’s and Ulcerative Colitis Association (EFCCA—Andrea Broggi, Policy Officer)
- The European Federation of Neurological Associations (EFNA—Manuela Messmer-Wullen, Board Member and Donna Walsh, Executive Director)
- The Stroke Alliance for Europe (SAFE—Manuela Messmer-Wullen, Board Member)
- Europa Donna—the European Breast Cancer Coalition (Roswitha Britz, Vice President)
- Europa Uomo—the European Prostate Cancer Coalition (Erik Briers, Ex officio Board Member)
- The Pelvic Pain Support Network (PPSN—Judy Birch, Head of Organisation)

From the ESR side, there are five “ex officio” representatives in the group:

- Chairperson of the ESR Communication and External Affairs Committee (vice-Chair of the Patient Advisory Group)
- ESR 2nd Vice-President
- ESR Quality, Safety and Standards Committee Chairperson
- ESR National Societies Committee Chairperson
- ESR Subspecialties and Allied Sciences Committee Chairperson

The group also includes a representative of the European Federation of Radiographer Societies (EFRS—Csaba Vandulek, President).

It has been decided that the group will be chaired by a patient representative and that the Chair of the Communication & External Affairs Committee of ESR will act as the Vice-Chairperson. During the first meeting, Nicola Bedlington, Secretary General of the European Patient Forum, has been designated by the other patient groups’ representatives as the Chairperson.

Some actions have been identified to fulfil the aims of the group, some work has started and the first activities have already been finalised:

## Collaboration on the International Day of Radiology (IDoR)

The ESR-PAG has been involved in the preparations of both IDoR 2013 and 2014. The book on thoracic radiology prepared for IDoR 2013 included interviews with selected patient representatives and some of the questions for the interviews with experts in thoracic radiology have been developed together with those patient representatives. In 2013, for the first time, a meeting on thoracic radiology held in Madrid during the celebrations of IDoR saw not only presentations from radiologists, but also a presentation on the point of view of patients with chronic pulmonary diseases. Furthermore, all patient associations have promoted the IDoR 2013 via their websites/newsletters.

In addition, the Group suggested focusing on “brain diseases” for the IDoR 2014, since this year has been the “European Year of the Brain”. The proposal was discussed and approved by the ESR and other co-organising Societies (RSNA and ACR) during the RSNA meeting in Chicago in December 2013. The European Federation of Neurological Associations (EFNA), in cooperation with the European Brain Council (EBC), has provided background information about the initiatives planned during 2014 to celebrate the “European Year of the Brain”, emphasising the role of radiology in clinical care and research in this field. A book on brain imaging has been prepared by the ESR Public Relations & Media Department, featuring interviews with selected patient representatives (Donna Walsh, Executive Director of the European Federation of Neurological Associations, and Manuela Messmer-Wullen, Stroke Alliance for Europe). Together with patients and patient representatives, the ESR Public Relations & Media Department developed a list of questions, which constituted the basis for interviews conducted with brain imaging experts from 19 European and seven South American countries.

## Improvement of the ESR patient information website

The “Services” section on the ESR website, dedicated to patients, provides information about a variety of radiological examinations, available for consultation. It has been decided to improve the information provided on the website.

This has started with the inclusion of four short articles entitled “A day in the life of…”. Two of these articles describe the experiences of a radiologist and of a radiographer during a standard workday; the other two relate to the feelings of two patients: one going to the hospital for a diagnostic examination and one coming for a radiation therapy treatment session. The scope of these articles is twofold: on the radiologist’s and radiographer’s side, we hope that the articles will improve the visibility of the radiologist’s and radiographer’s day-to-day-work; on the patient’s side the articles are meant to explain how patients feel when they come to the hospital and what they expect from the radiologist.

Radiological studies are often regarded as “scary” or, on the contrary, as non-relevant examinations. Thus, a link to the ESR EuroSafe Imaging campaign has been added to the website to help patients understand the issues in radiation protection that the ESR is currently dealing with.

In addition, to try to overcome language barriers within Europe, links to the websites of European hospitals and national radiological societies have been added, so patients can download information directly in their own languages.

The website also includes information on the activities promoted each year by the group during the International Day of Radiology as well as on the results of the Alliance for MRI initiative (as examples of good collaboration).

## Involvement of patient representatives in ESR initiatives

### At ECR

Involvement of the ESR Patient Advisory Group has been deeply imbedded into the ECR 2014 programme. The Chairperson, Nicola Bedlington participated in the press conference held before the congress, was involved in the launch of the ESR EuroSafe Imaging Campaign and gave a talk on “Radiation risk: a patient’s perspective” during the session on: “Good radiation and bad radiation? How to assess and communicate radiation risk to patients and referring physicians”. Furthermore, E. Briers presented “The patient’s perspective” during the session on Patient-Centred Radiology and Economics, and J. Brandstätter talked about “Interactions between patients and radiologists through social media” during the Professional challenges session dedicated to the role of social media in radiology.

The Patient Advisory Group’s involvement in the programme of ECR 2015 is currently under preparation, but promises to be very important. In fact, its Chairperson will be once again present at the press conference at the opening of the conference, but this time there will be two full sessions directly organised by the group: one entitled “Communicating the results of radiological studies to patients: from high-tech to human-touch imaging” and one dedicated to “The challenges of providing true patient-centred care—moving forward together”. The latter will present a driver diagram developed together by patient groups and the ESR Audit and Standards Subcommittee aimed at providing a framework on how to establish an organisation to ensure patient-centred care within Radiology Departments. Once again, as in 2014, there will be the possibility to listen directly to patients’ or patient representatives’ voices. Hearing discussions on radiological topics from their point of view will surely open the radiologist’s perspective and help understand how to meet the patient’s expectations.

### Other initiatives

Since the formation of the Patient Advisory Group, other ESR initiatives saw the active contribution of patients. During the Management in Radiology (MIR) 2013 meeting held in Barcelona, patient representative T. Szelagowski (EPF) gave a talk on “eHealth—patients’ perspective” and during a high-level roundtable discussion on the “Role of Imaging in Personalised Medicine” organised by the ESR and the European Alliance for Personalised Medicine (EAPM), which took place at the European Parliament in Brussels in October 2013, the patients’ point of view was presented by patient representative Erik Briers, Ex Officio Board Member of Europa Uomo—the European Prostate Cancer Coalition.

Another event at the European Parliament in Brussels on the launch of the “European Action Plan for Medical Imaging to improve quality of care and patient safety” took place in November 2014, including contributions of members of the Patient Advisory Group.

During the ECR 2014, the EuroSafe Imaging Campaign was launched. This flagship initiative of the ESR aims at promoting quality and safety in medical imaging and Manuela Messmer-Wullen, representing EFNA and SAFE at the Patient Advisory Group has been assigned as a member of the EuroSafe Imaging Steering Committee.

The Alliance for MRI, founded by the ESR, and including Members of the European Parliament (MEPs), patient groups and the scientific community, with the aim to ensure unimpeded access to MRI technology for all patients across the EU, was successfully concluded in early summer 2013. It serves as good example for the importance of combined efforts between patient organisations and the scientific community in European Affairs. Additional fields of collaboration are currently being explored.

## Involvement of ESR in Patient Groups’ initiatives

The link established between the ESR and Patient Groups with the launch of the ESR Patient Advisory Group has been announced in the European Patient Forum (EPF) newsletter and described as “a new model for working with medical Societies” [4]. In fact, patient-doctor cooperations have usually emerged within disease-specific societies and groups, and this is the first time a large, specialty-based medical association has started working with several patient groups.

Every 5 years, shortly before the elections for the European Parliament, the EPF launches a campaign to encourage politicians and policy-makers to commit to a healthier Europe, where patients are part of the solution to make the health systems of European Nations more effective and quality-oriented. The campaign is not aimed at sustaining any political party, but is supporting equitable access to healthcare, patient empowerment and patient involvement in health affairs management. This initiative, in which Patient Groups, many individuals from all over Europe and many members of the European Parliament have been engaged, has been officially supported by the ESR, which was the first medical professional organisation to join it.

These efforts aim at the development of bi-directional relationships, with advantages for both parts. Radiologists and patients, in fact, need to be “educated” to improve communication.

In an era of “personalised” medicine, in which decisions and practice are tailored to the individual patient in whatever ways possible (systematic use of genetic or other personal information to optimise the patient’s preventative and therapeutic care), radiology is also becoming “personalised” [5]. On the other hand, the concept of “patient-centred” medicine is developing (care designed to suit patient’s and not doctor’s needs, shared decision-making, patient governance, mutual commitment to quality and health outcomes) [6] and “patient-centred” radiology is evolving as well [7, 8].

These two concepts seem to be two sides of the same coin. Personalised medicine (and personalised radiology) works at a physical and biological level; patient-centred medicine pays attention to the psychological, behavioural and relational aspects of medical (and radiological) work. Both are essential elements for good outcomes in medical (and radiological) practice.

## Conclusions

In summary, the ESR strongly believes that close collaboration with patient organisations is beneficial for both stakeholders. On the one hand, this initiative helps radiologists to better understand their patients’ needs, to adapt their practice accordingly and to improve their communication skills. This will facilitate the perception of radiologists as clinicians, and will contribute towards overcoming the poor visibility of the specialty [9]. On the other hand, close collaboration with professionals will allow patients to obtain a better understanding of the work of all the people involved in a radiological department, of the advantages and limitations of imaging and of what can be expected from a radiological examination or an imaging-guided interventional procedure. Thus, it should be clear that this collaborative effort is not simply limited to improving patient satisfaction; the ultimate goal is to enhance the faith and trust that patients place in the medical imaging profession and all the radiology professionals involved. At the ESR we are profoundly convinced that well-informed patients can become actively involved in their healthcare, and this is an important element towards improving the partnership between patients and radiology professionals. Together, we are working towards achieving this shared ambition, and the ESR-PAG is leading the way.
